# Characteristics and Prognosis of Wild-Type Transthyretin Amyloid Cardiomyopathy Patients Diagnosed Before 65 Years Old

**DOI:** 10.1016/j.jacadv.2025.102354

**Published:** 2025-11-21

**Authors:** Damien Guijarro, Jean-Christophe Eicher, Mélanie Bézard, Nicolas Piriou, François Sauer, François Roubille, Jérôme Costa, Patricia Réant, Erwan Donal, Fabrice Bauer, Arnaud Bisson, Océane Bouchot, Eve Cariou, Olivier Lairez, Pierre-Yves Courand, Charlotte Dagrenat, Jean-Pierre Gueffet, Gilbert Habib, Julien Jeanneteau, Léa Margerit, Silvia Oghina, Romain Trésorier, Mounira Kharoubi, Thibaud Damy

**Affiliations:** a Department of Cardiology, Geneva University Hospitals, Geneva, Switzerland; b Department of Cardiology, CHU de Dijon Bourgogne, Hôpital François Mitterrand, Dijon, France; c Department of Cardiology, Referral Center for Cardiac Amyloidosis, Filiere Cardiogen, GRC Amyloid Research Institute All at APHP CHU Henri Mondor, Créteil, France; d Heart’s Foundation, Vincennes, France; e Nantes Université, CHU Nantes, INSERM, Cardiology Department, Institut du thorax, Nantes, France; f Department of Cardiology, Nouvel Hôpital Civil – Hopitaux Universitaires de Strasbourg, Strasbourg, France; g Department of Cardiology, CHU de Montpellier, Montpellier, France; h Department of Cardiology, CHU Robert Debré, Reims, France; i Bordeaux University Hospital Center, Bordeaux University, Bordeaux, France; j Université de Rennes, CHU Rennes, Service de Cardiologie Inserm, Rennes, France; k Department of Cardiology, CHU Bicêtre, Le Kremlin Bicêtre, France; l Department of Cardiology, CHRU de Tours, Tours, France; m Department of Cardiology, Centre Hospitalier Annecy Genevois, Epagny Metz-Tessy, France; n Department of Cardiology, Cardiac Imaging Centre, University Hospital of Toulouse, Toulouse, France; o Department of Cardiology, Hôpital de la Croix Rousse et Lyon Sud, Hospice Civil de Lyon, Lyon, France; p Department of Cardiology, Centre Hospitalier Haguenau, Hagueneau, France; q Department of Cardiology, Hôpital Privé Confluent, Nantes, France; r Department of Cardiology, Assistance Publique Hôpitaux de Marseille, CHU de La Timone, Marseille, France; s Department of Cardiology, Clinique Saint Joseph, Trelaze, France; t Department of Cardiology, Groupe Hospitalier Mutualiste de Grenoble, Grenoble, France; u Department of Cardiology, CHU de Clermont Ferrand, Clermont Ferrand, France; v Clinical Epidemiology and Ageing (CEpiA) at Henri Mondor University Hospital and Institut National de la Santé et de la Recherche Médicale (INSERM) U955, Institut Henri Mondor University Hospital, Créteil, France

**Keywords:** amyloidosis, diagnosis, prognosis, transthyretin

## Abstract

**Background:**

Guidelines recommend screening for transthyretin amyloid cardiomyopathy (ATTR-CM) after age 65 years, yet some patients are diagnosed earlier.

**Objectives:**

The purpose of this study was to compare the proportion, clinical characteristics, and prognosis of wild-type ATTR-CM diagnosed ≤65 years (ATTRwt-Yy) with those diagnosed >65 years (ATTRwt-O).

**Methods:**

Data from the HEAR (Healthcare European Amyloidosis Registry), a multicenter, noninterventional, longitudinal registry, were analyzed. Patients were categorized by age at diagnosis: ATTRwt-Yy (≤65 years) and ATTRwt-O (>65 years). ATTRwt-O patients were further classified by onset of first cardiac symptom: ATTRwt-Oy (≤65 years) and ATTRwt-Oo (>65 years).

**Results:**

From July 2021 to May 2024, 3,980 ATTR patients were enrolled; 1,417 had ATTRwt-CM with documented symptom onset. Among them, 67 (4.7%) were ATTRwt-Yy, 111 (7.8%) ATTRwt-Oy, and 1,239 (87.4%) ATTRwt-Oo. Diagnostic delays were 0.65, 20.58, and 0.77 years, respectively (*P* < 0.001). Heart failure signs at presentation were seen in 34.9% of ATTRwt-Yy, 8.1% of ATTRwt-Oy, and 30.2% of ATTRwt-Oo (*P* < 0.001). ATTRwt-Yy patients had more extracardiac manifestations, notably osteoarticular disease, whereas rhythm disturbances predominated in ATTRwt-Oy. Median follow-up from diagnosis was 36.2, 23.6, and 22.4 months, respectively. ATTRwt-Yy patients had better survival after diagnosis compared to ATTRwt-O patients.

**Conclusions:**

ATTRwt-CM diagnosed before 65 years shows a distinct phenotype highlighting the need for tailored diagnostic and management strategies.

Transthyretin amyloid cardiomyopathy (ATTR-CM) is a progressive infiltrative disease that is either of genetic origin, when associated with a pathogenic mutation on the transthyretin gene, or of wild-type origin (ATTRwt-CM). To date, the pathophysiological mechanisms leading to fibril formation and their deposition in tissues in wild-type forms of the disease remain largely unknown.^1^

For decades, ATTRwt-CM has been considered as a rare disease affecting mainly patients over the age of 70 years: with a median age at diagnosis of 73.0 years (Q1-Q3: 69.5-78.2).^2^ An historical study, reported in 1983, on explanted hearts of 85 consecutive patients aged 80 years or older, found that 25% had histologic evidence of transthyretin deposits.^3^ The most frequent clinical scenario, at diagnosis, is increased cardiac wall thickness with either heart failure or red flag signs/symptoms in men older than 65 years or in women older than 70 years.^4^ Consequently, the European Society of Cardiology (ESC) guidelines in 2021^5^ and then in 2023^6^ set the age cutoff of 65 years for suspecting ATTR-CM.

The epidemiology of ATTRwt-CM has evolved in recent years. Following the development of the diagnostic algorithm^5^ and the increased reliability of noninvasive diagnostic methods,^5^^,^^7^ the prevalence of ATTR-CM has continued to increase.^8^^,^^9^ Moreover, ATTRwt-CM is an emerging etiology for numerous degenerative heart diseases, including heart failure (either with preserved or reduced ejection fraction), valvulopathy, and conductive disorders.^10^ An Italian systematic screening study found an estimate ATTRwt-CM prevalence of 0.46% of patients aged 65 to 90 years old and provides evidence that ATTRwt-CM can no longer be considered a rare disease according to European and American definitions.^11^

With the improved screening and diagnosing of ATTRwt-CM by physicians, a few but not insignificant number of patients younger than the 65-year age cutoff, suggested by the ESC,^5^^,^^6^ have been diagnosed.^8^^,^^12^^,^^13^ However, the characteristics and outcomes in these young patients have not been specifically studied. Currently, the published data are limited, including isolated case reports of exceptionally young patients with poor prognosis.^13^^,^^14^

The aim of this study was to assess the proportion, characteristics, and prognosis of patients diagnosed with ATTRwt-CM at or before the age of 65 years (ATTRwt-Yy group) in the HEAR (Healthcare European Amyloidosis Registry) compared to older patients (ATTRwt-O).

## Methods

### The Healthcare European Amyloidosis Registry

HEAR was designed as a French, multicenter, observational registry to collect data of patients with suspected or confirmed cardiac amyloidosis (CA). Participating centers are requested to enroll all consecutive eligible patients. Patients with all types of CA are eligible. The registry collects data both retrospectively and prospectively. In addition to patients with a confirmed diagnosis of CA, the registry collects data from patients with suspected CA, not subsequently confirmed. The collected data include, but are not limited to, demographic data, quality of life data, imagery, as well as clinical, biological, and safety data. The main objective of the registry is to collect data concerning the natural evolution of amyloidosis and health care management throughout the patients’ life from when CA is suspected. The collected data are used to conduct various studies concerning the evolution and management of patients with CA. The study adheres to French and European Law (classified as MR004 [reference 2223041v0]). HEAR was approved by the ethics committee (“Comité de protection des personnes du Sud-Ouest et Outre-Mer 2”; N2019-A02010-57) and is registered in the clinicaltrial.gov database (NCT05101304).

### Amylo-young study

#### Study design

The Amylo-Young study was designed to investigate the characteristics and survival outcomes of patients with confirmed ATTRwt-CM according to their age at diagnosis and onset of first cardiac symptoms. The confirmed diagnosis of ATTRwt-CM was based on the current guidelines. ATTRwt-CM diagnosis could be established either invasively by a biopsy-proven diagnosis or noninvasively by a positive bone scintigraphy (Perugini grade II or III) and without evidence of monoclonal gammopathy. The classification of ATTRwt-CM as wild type was based on the absence of a transthyretin genetic mutation. Patients with hereditary ATTR or with an unknown genetic status were not included in the study.

#### Study cohorts

The patients included in the study were those enrolled in the HEAR with a confirmed diagnosis of ATTRwt-CM and were classified according to the age at confirmed diagnosis (date of the biopsy or of the bone scintigraphy), as either ≤65 years old (ATTRwt-Y group) or >65 years (ATTRwt-O group) at diagnosis. Secondly, patients were classified by their age of first cardiac symptoms before either ≤65 years old (y) or >65 years (o). Overall, the analysis was performed in 3 groups of patients all with confirmed diagnosis of ATTRwt-CM: those diagnosed when ≤65 years old (ATTRwt-Yy group), those diagnosed when >65 years old with early signs and symptoms (ATTRwt-Oy group), and those diagnosed when >65 years old without early signs and symptoms (ATTRwt-Oo group) (Central Illustration). Indeed, patients with early cardiac signs and symptoms (ATTRwt-Oy), diagnosed later, may have a disease phenotype that favors a delayed diagnosis. Thus, these patients were analyzed separately.

**Central Illustration:**
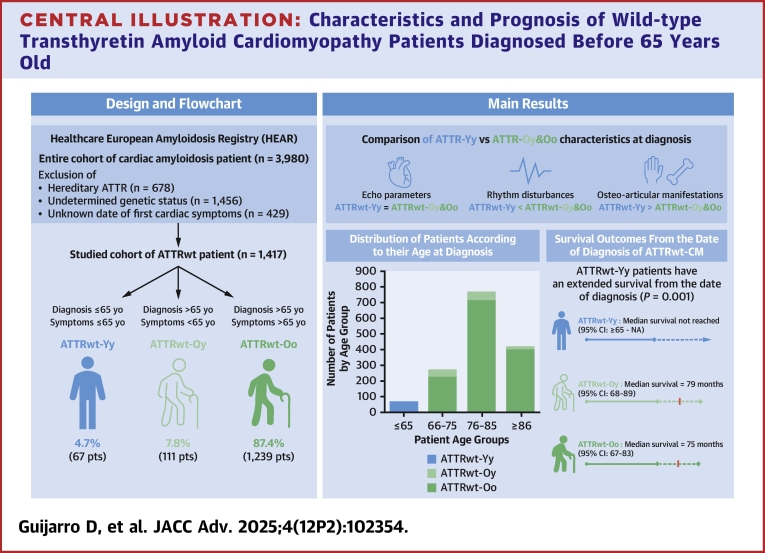
**Characteristics and Prognosis of Wild-type Transthyretin Amyloid Cardiomyopathy Patients Diagnosed Before 65 Years Old** Among 1,417 patients with ATTRwt-CM, 4.7% were diagnosed at ≤65 years of age (ATTRwt-Yy group). These patients displayed distinct clinical characteristics and demonstrated longer survival after diagnosis compared with older patients. Abbreviations as in Figure 1, Figure 2, Figure 3, Figure 4.

#### Study objectives

The main objective of the study was to identify and estimate the proportion of patients in HEAR with a diagnosis of ATTRwt-CM before or at the age of 65 years (patients in the ATTRwt-Yy group) and to describe their characteristics. Furthermore, the study aimed to compare the characteristics and survival of patients in the ATTRwt-Yy group to those diagnosed after the age of 65 years, in the ATTRwt-Oo and ATTR-Oy groups.

### Statistical methods

The results of the analysis are reported using descriptive statistics. Continuous data are presented as mean with SD or median with 25th-75th percentiles (Q1-Q3). Categorical data are presented as frequencies with percentages. The normality of the distribution of continuous data was verified using the Shapiro-Wilk test and the heteroskedasticity using the Levene’s test. The diagnostic delay is defined as the time between the age of first cardiac symptoms and the age of ATTRwt-CM diagnosis. Outcomes based on continuous data were compared using analysis of variance, Welch's analysis of variance, or the Kruskal-Wallis test depending on the data distribution. Outcomes based on categorical data were compared using either chi-squared or Fisher exact tests. Statistical tests were not performed when fewer than 5 patients were present in at least 2 groups. NYHA functional class was compared between the 3 groups (ATTRwt-Yy, ATTRwt-Oy, ATTRwt-Oo). Because the number of patients in NYHA IV was very low in ATTRwt-Yy and ATTRwt-Oy (n = 1 each), NYHA III and IV were combined for statistical analysis. A 2-sided *P* value <0.05 was considered statistically significant. Survival was assessed using the Kaplan-Meier method. Time 0 on the Kaplan-Meier plots are the date of ATTRwt-CM diagnosis. Groups are compared using the log-rank test and presented using the median time since diagnosis with 95% CI. Statistical analysis was performed using EasyMedStat (version 3.35.1).

## Results

### Study populations and proportion

**Figure 1:**
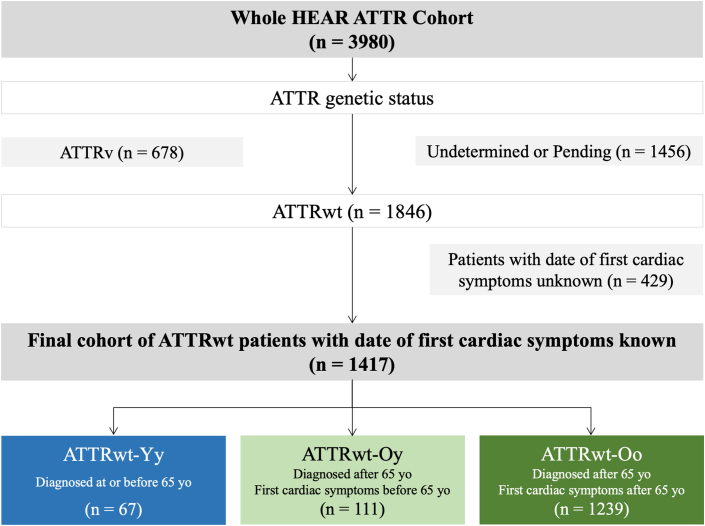
**Flowchart of the Study Groups** ATTR = transthyretin amyloidosis; ATTRv = hereditary transthyretin amyloidosis; ATTRwt = wild-type transthyretin amyloidosis; ATTRwt-Oo = patients diagnosed with ATTRwt after the age 65 years and without cardiac symptoms reported before the age of 65 years; ATTRwt-Oy = patients with ATTRwt diagnosed after the age 65 years and with cardiac symptoms reported at or before the age of 65 years; ATTRwt-Yy = patients diagnosed with ATTRwt at or before the age 65 years and with cardiac symptoms reported at or before the age of 65 years; HEAR = Healthcare European Amyloidosis Registry.

On May 24, 2024, when the data were extracted, of the 5,730 patients enrolled in the HEAR, 3,980 had been diagnosed with ATTR-CM, of which 1846 had confirmed ATTRwt-CM. Among the patients with ATTRwt-CM, only 1,417 had a known date of first cardiac symptom and were included in this study. Among these patients, 67 (4.7%) were classified as ATTRwt-Yy and 1,350 (95.3%) as ATTRwt-O (Figure 1, Central Illustration). Of the 1,350 patients in the ATTRwt-O group, 111 (8.2%) were classified as ATTRwt-Oy and 1,239 (91.8%) as ATTRwt-Oo. Thus, the proportion of patients, in the HEAR, with ATTRwt-CM diagnosed at or before the age of 65 years was 67 of 1,417 patients (4.7%).

### ATTRwt-CM diagnosis and first cardiac and extracardiac symptoms

**Figure 2:**
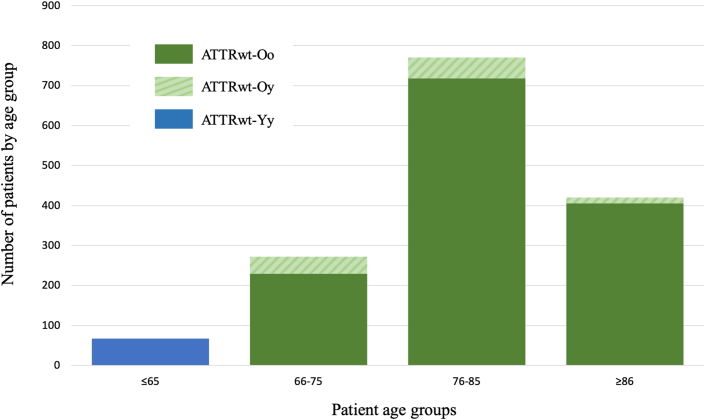
**Distribution of 3 Patient Groups by Age at ATTR Diagnosis** Abbreviations as in Figure 1.

The age at diagnosis, first cardiac symptom, and scintigraphy, as well as the diagnostic delay, (overall and according to the study groups) are shown in Table 1. The median diagnostic delay was 0.7 years (Q1-Q3: 0.1-2.0) in the ATTRwt-Yy group and 0.8 years (Q1-Q3: 0.2-3.2) in the ATTRwt-Oo group. The delay was significantly longer in the ATTRwt-Oy group: 20.6 years (Q1-Q3: 12.0-22.5), *P* < 0.001. The distribution of patients by age at diagnosis and by age at first cardiac symptoms is shown graphically in Figure 2.

**Table 1:** Diagnostic and First Symptoms Characteristics of the Overall Cohort and According to ATTRwt Groups

	Overall Cohort (N = 1,417)	ATTRwt-Yy (n = 67)	ATTRwt-Oy (n = 111)	ATTRwt-Oo (n = 1,239)	*P* Value
Age at CA diagnosis (y), (±SD)	81.0 (±9.2)	62.5 (±4.6)	78.3 (±6.8)	82.5 (±6.0)	<0.001
Age of first CV symptoms (y), (±SD)	77.4 (±10.1)	61.0 (±5.4)	60.9 (±4.8)	79.9 (±6.6)	<0.001
Age at scintigraphy (y), (±SD)	80.0 (±13.6)	61.9 (±4.4)	78.1 (±6.7)	81.9 (±10.1)	<0.001
Diagnosis delay (y), median (Q1-Q3)	1.0 (0.2-4.3)	0.7 (0.1-2.0)	20.6 (12.0-22.5)	0.8 (0.2-3.2)	<0.001
First cardiac symptoms					
Angina, n (%)	12 (0.9)	1 (1.6)	5 (4.5)	6 (0.5)	-
Atrial arrhythmias, n (%)	366 (25.8)	8 (12.7)	44 (39.6)	314 (25.4)	<0.001
Aortic stenosis, n (%)	35 (2.5)	0 (0.0)	3 (2.7)	32 (2.6)	-
Chest pain, n (%)	37 (2.6)	2 (3.2)	5 (4.5)	30 (2.4)	ns
Dyspnea/edema, n (%)	404 (28.5)	22 (34.9)	9 (8.1)	373 (30.2)	<0.001
Pulmonary embolism, n (%)	6 (0.4)	0 (0.0)	1 (0.9)	5 (0.4)	-
Bone scintigraphy uptake, n (%)	73 (5.2)	7 (11.1)	0 (0.0)	66 (5.3)	-
Arterial hypertension, n (%)	21 (1.5)	0 (0.0)	9 (8.1)	12 (1.0)	<0.001
Cardiac hypertrophy, n (%)	342 (24.1)	17 (27.0)	19 (17.1)	306 (24.7)	0.02
Palpitations, n (%)	10 (0.7)	1 (1.6)	3 (2.7)	6 (0.5)	-
Syncope, n (%)	16 (1.1)	1 (1.6)	0 (0.0)	15 (1.2)	-
Elevated troponin level, n (%)	11 (0.8)	0 (0.0)	1 (0.9)	10 (0.8)	-
Conductive disorders, n (%)	56 (4.0)	3 (4.8)	10 (9.0)	43 (3.5)	0.002
Valves thickening, n (%)	2 (0.1)	0 (0.0)	0 (0.0)	2 (0.2)	-
Other, n (%)	20 (1.4)	1 (1.6)	2 (1.8)	17 (1.4)	-
First extracardiac symptoms					
Stroke/TIA, n (%)	36 (2.5)	0 (0.0)	5 (4.6)	31 (2.6)	ns
Deterioration of general condition, n (%)	6 (0.4)	0 (0.0)	0 (0.0)	6 (0.5)	-
Lumbar spinal stenosis, n (%)	47 (3.3)	3 (4.9)	4 (3.6)	40 (3.3)	-
Carpal tunnel syndrome, n (%)	285 (20.1)	25 (41.0)	22 (20.0)	238 (19.8)	<0.001
Diarrhoea/Constipation, n (%)	6 (0.4)	0 (0.0)	2 (1.8)	4 (0.3)	-
Muscular pain, n (%)	3 (0.2)	0 (0.0)	0 (0.0)	3 (0.3)	-
Dupuytren's contracture, n (%)	29 (2.1)	1 (1.6)	1 (0.9)	27 (2.3)	-
Dysphonia, n (%)	3 (0.2)	0 (0.0)	0 (0.0)	3 (0.3)	-
Periorbital ecchymosis, n (%)	1 (0.1)	0 (0.0)	0 (0.0)	1 (0.1)	-
Monoclonal gammopathy, n (%)	9 (0.6)	0 (0.0)	0 (0.0)	9 (0.8)	-
Postural hypotension, n (%)	21 (1.5)	0 (0.0)	3 (2.7)	18 (1.5)	-
Renal failure, n (%)	16 (1.1)	0 (0.0)	2 (1.8)	14 (1.2)	-
Nail lesions, n (%)	4 (0.3)	0 (0.0)	1 (0.9)	3 (0.3)	-
Macroglossia, n (%)	5 (0.4)	0 (0.0)	0 (0.0)	5 (0.4)	-
Neuropathy, n (%)	39 (2.8)	6 (9.8)	2 (1.8)	31 (2.6)	0.01
Knee replacement, n (%)	39 (2.8)	2 (3.3)	1 (0.9)	36 (3.0)	-
Hip replacement, n (%)	172 (12.1)	4 (6.6)	15 (13.6)	153 (12.7)	ns
Loss of weight, n (%)	3 (0.2)	0 (0.0)	0 (0.0)	3 (0.3)	-
Cutaneous purpura, n (%)	2 (0.1)	0 (0.0)	0 (0.0)	2 (0.2)	-
Deafness, n (%)	112 (7.9)	1 (1.6)	12 (10.9)	99 (8.2)	ns
Erectile dysfunction, n (%)	1 (0.1)	0 (0.0)	0 (0.0)	1 (0.1)	-
None, n (%)	222 (15.7)	4 (6.6)	13 (11.8)	205 (17.1)	0.03

ATTRwt = wild-type transthyretin amyloidosis; ATTRwt-Oo = patients with ATTRwt diagnosed after the age of 65 years without first cardiac symptoms at or before 65 years of age; ATTRwt-Oy = patients with ATTRwt diagnosed after the age of 65 years with first cardiac symptoms at or before 65 years of age; ATTRwt-Yy = patients with ATTRwt diagnosed at or before the age of 65 years; CA = cardiac amyloidosis; CV = cardiovascular; Q1-Q3 = 25th-75th percentiles; ns = nonsignificant; TIA = transient ischemic attack.

In terms of first cardiac symptoms, atrial arrhythmia occurred in 12.7% of patients in the ATTRwt-Y, 25.4% in the ATTRwt-Oo, and 39.6% in the ATTRwt-Oy group (Table 1). The relative occurrences of the other first cardiac symptoms were similar in the ATTRwt-Yy and ATTRwt-Oo groups. In contrast, patients in the ATTRwt-Oy group had a different profile with relatively more arterial hypertension and conductive disorders, and fewer dyspnea/edema and cardiac hypertrophy as first cardiac events. Regarding the first extracardiac symptoms, carpal tunnel syndrome (CTS) was more frequent in the ATTRwt-Yy group, 41.0% compared to 20.0% in the ATTRwt-Oy group and 19.8% in the ATTRwt-Oo group (Table 1). Similarly, neuropathy occurred more frequently in the ATTRwt-Yy group. In contrast, fewer patients in the ATTRwt-Yy group had hip replacement and deafness as first extracardiac symptom compared to the patients in the ATTRwt-O groups.

### Patient characteristics

**Figure 3:**
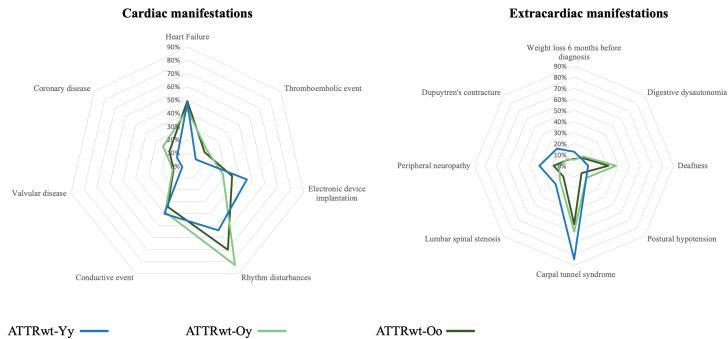
**Spider Charts of Cardiac and Extracardiac Events by Study Group** Abbreviations as in Figure 1.

The phenotypes of patients at inclusion in the registry, overall and in the groups, are described in Table 2 and shown graphically in Figure 3. Compared to patients in the ATTRwt-Oo group, significantly fewer patients in the ATTRwt-Yy group have rhythm disorders and deafness (Table 2). However, significantly more had weight loss within the 6 months before diagnosis, CTS, lumbar spinal stenosis, peripheral neuropathy, and Dupuytren’s contractures. In terms of electrocardiography parameters, significantly fewer patients in the ATTRwt-Yy group had atrial fibrillation and the PR interval and QRS were significantly shorter. Patients in the ATTRwt-Yy group also had significantly higher glomerular filtration rate (Cockcroft-Gault formula) and high-sensitivity troponin-T levels, but significantly lower N-terminal pro-B-type natriuretic peptide levels. Morphological and functional echocardiographic data were similar between the groups.

**Table 2:** Demographic, Clinical, Biological, Cardiac Imaging Baseline Characteristics at Diagnosis of the Overall Cohort and According to ATTRwt Groups

	Overall Cohort (N = 1,417)	ATTRwt-Yy (n = 67)	ATTRwt-Oy (n = 111)	ATTRwt-Oo (n = 1,239)	*P* Value
Male, n (%)	1,215 (85.7)	58 (86.6)	105 (94.6)	1,052 (84.9)	0.019
Hypertension, n (%)	854 (60.3)	34 (65.4)	73 (76.8)	747 (73.2)	0.32
Diabetes, n (%)	258 (18.2)	8 (15.4)	21 (22.1)	229 (22.4)	0.49
Dyslipidemia, n (%)	522 (36.8)	21 (40.4)	43 (45.3)	457 (44.8)	0.82
Tobacco abuse, n (%)	332 (23.4)	18 (34.6)	35 (36.8)	279 (27.3)	0.087
Cardiac manifestations					
Heart failure, n (%)	600 (42.3)	24 (48.0)	45 (42.5)	531 (49.0)	0.44
Thromboembolic event, n (%)	204 (14.4)	4 (8.0)	20 (18.9)	180 (16.6)	0.22
Electronic device implantation	426 (30.0)	23 (46.0)	29 (27.4)	374 (34.5)	0.070
Rhythm disturbances, n (%)	876 (61.8)	27 (54.0)	88 (83.0)	761 (70.2)	<0.001
Conductive event, n (%)	432 (30.5)	20 (40.0)	41 (38.7)	371 (34.2)	0.48
Valvular disease, n (%)	130 (9.2)	2 (4.0)	12 (11.3)	116 (10.7)	0.31
Coronary disease, n (%)	218 (15.4)	5 (10.0)	25 (23.6)	188 (17.4)	0.098
Extracardiac manifestations					
Weight loss before diagnosis, n (%)	87 (6.1)	8 (12.7)	6 (5.6)	73 (6.1)	0.002
Digestive dysautonomia, n (%)	143 (10.1)	6 (9.38)	13 (11.8)	124 (10.1)	0.74
Deafness, n (%)	433 (30.6)	8 (12.7)	42 (37.8)	383 (31.0)	0.003
Postural hypotension, n (%)	144 (10.2)	10 (15.6)	17 (15.5)	117 (9.5)	0.098
Carpal tunnel syndrome, n (%)	771 (54.4)	54 (84.4)	66 (59.5)	651 (52.8)	<0.001
Lumbar spinal stenosis, n (%)	205 (14.5)	15 (23.4)	20 (18.0)	170 (13.8)	0.014
Peripheral neuropathy, n (%)	262 (18.5)	20 (31.3)	15 (13.5)	227 (18.5)	0.036
Dupuytren’s contracture, n (%)	116 (8.2)	14 (21.9)	9 (8.3)	93 (7.7)	<0.001
Clinical and paraclinical data					
Body mass index, kg/m^2^	25.97 ± 4.4	27.15 ± 5.2	25.85 ± 3.4	25.93 ± 4.4	0.11
NYHA I, n (%)	244 (17.2)	17 (29.3)	22 (21.8)	205 (19.9)	0.088
NYHA II, n (%)	666 (47.0)	28 (48.3)	59 (58.4)	579 (56.2)	0.07
NYHA III, IV n (%)	259 (18.3)	13 (22.4)	20 (19.8)	246 (23.9)	0.89
Heart rate, beats/min	75.8 ± 16.1	75.7 ± 15.4	77.3 ± 20.1	75.6 ± 15.7	0.94
Systolic blood pressure, mm Hg	135.0 ± 19.9	132.8 ± 19.3	135.5 ± 21.4	135.1 ± 19.8	0.70
Electrocardiogram					
Atrial fibrillation, n (%)	394 (27.8)	6 (11.5)	42 (44.7)	346 (38.8)	<0.001
PR interval, ms	207 ± 99	185 ± 33	199 ± 59	209 ± 106	0.037
QRS, ms	118 ± 34	108 ± 32	131 ± 50	117 ± 32	0.001
Low QRS voltage, n (%)	176 (12.4)	5 (26.32)	11 (50.0)	160 (46.0)	0.22
Biology					
Cockcroft-Gault GFR, mL/min	58.7 ± 26.0	87.9 ± 32.0	70.0 ± 30.7	51.3 ± 18.3	<0.001
hs-TnT, ng/L	68.2 ± 69.0	73.7 ± 156.1	65.8 ± 41.0	68.1 ± 63.1	0.029
NT-proBNP, ng/L	3,414 ± 4,164	2,461 ± 4,581	3,118 ± 3,587	3,492 ± 4,183	<0.001
Echocardiography					
IVSd, mm	17.0 ± 3.5	17.1 ± 3.4	15.9 ± 2.7	16.7 ± 3.5	0.055
PWd, mm	15.2 ± 3.4	15.1 ± 3.5	14.5 ± 2.9	14.9 ± 3.4	0.46
LVEF, %	52.7 ± 12.1	52.9 ± 13.2	51.8 ± 12.8	52.8 ± 12.0	0.89
GLS, %	−12.0 ± 4.4	−11.5 ± 4.6	−12.5 ± 5.4	−12.0 ± 4.2	0.71
Bone scintigraphy					0.014
Cardiac hyperfixation without score	217 (15.3)	25 (41.7)	19 (21.1)	173 (18.3)	
Perugini score 0	6 (0.4)	0 (0.0)	0 (0.0)	6 (0.6)	
Perugini score 1	30 (2.1)	0 (0.0)	2 (2.2)	28 (3.0)	
Perugini score 2	443 (31.3)	16 (26.7)	40 (44.4)	387 (41.0)	
Perugini score 3	399 (28.2)	19 (31.7)	29 (32.2)	351 (37.1)	
Treatment					
Tafamidis, n (%)	1,309 (92.4)	60 (89.6)	103 (92.8)	1,146 (92.5)	0.097

Data are presented as count (percentage) or mean (±SD).

GFR = glomerular filtration rate; GLS = global longitudinal strain; hs-TnT = high-sensitivity troponin-T; IVSd = interventricular septum thickness at end diastole; LVEF = left ventricular ejection fraction; NT-proBNP = N-terminal pro-B-type natriuretic peptide; PWd = posterior wall thickness at end diastole; other abbreviations as in Table 1.

### Survival outcomes

Survival was assessed after a median follow-up, from the date of diagnosis, in the overall population of 22.8 months (Q1-Q3: 12.0-36.5): 36.2 months (Q1-Q3: 18.9; 61.1) in the ATTRwt-Yy group, 23.6 months (Q1-Q3: 14.4; 34.3) in the ATTRwt-Oy group, and 22.4 months (Q1-Q3: 11.7-34.5) in the ATTRwt-Oo group.

**Figure 4:**
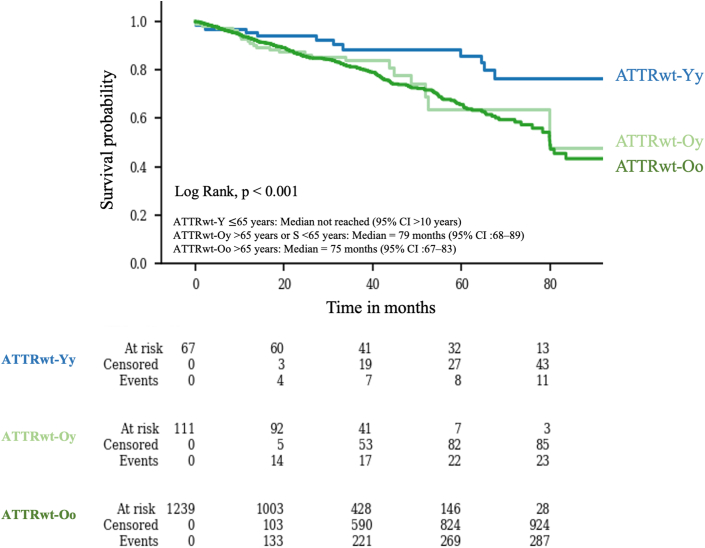
**Kaplan-Meier Survival Curves From Diagnosis for 3 ATTRwt-CM Cohorts** Abbreviations as in Figure 1.

The analysis of survival in the study groups is shown in Figure 4. The overall survival from the date of diagnosis was significantly longer in the ATTRwt-Yy group. In the Kaplan-Meier analysis, the ATTRwt-Oo group showed a median survival of 75 months ([95% CI: 67-83]). The ATTRwt-Oy group had a median survival of 79 months ([95% CI: 68-89]. For ATTRwt-Yy, the median survival was not reached; notably, the lower 95% confidence bound was ≥65 months, with the upper bound not estimable. These results indicate separation among the 3 groups, with ATTRwt-Yy demonstrating the most favorable early survival profile (*P* = 0.001). Thus, patients diagnosed with ATTRwt-CM at or before the age of 65 years (in the ATTRwt-Yy group) have an extended survival from the date of diagnosis.

## Discussion

The Amylo-Young study identified a group of ATTRwt-CM patients in the HEAR diagnosed at or before the age of 65 years. The study shows a non-negligible proportion of 4.7% of these younger patients within the studied ATTRwt-CM cohort. These patients have distinct characteristics and survival outcomes. Patients with early diagnosis (at or before the age of 65 years) had more extracardiac signs and symptoms (CTS, hip replacements, peripheral neuropathy, and Dupuytren’s contractures) of ATTR at diagnosis and had an extended survival from the date of diagnosis compared to those diagnosed when older. We also identified a group of patients (7.8% of the overall cohort) diagnosed after the age of 65 years but with early signs and symptoms of ATTRwt-CM. These patients also had distinct profiles, in terms of their first signs and symptoms of ATTR-CM and survival outcomes.

### Proportion of young patients diagnosed with ATTR-CM

The 4.7% proportion of patients diagnosed with ATTRwt-CM at or before the age of 65 years is coherent with the age distribution at enrollment observed in the THAOS (the Transthyretin Amyloidosis Outcomes Survey) registry.^13^ Of the 1,251 patients with ATTRwt-CM in the THAOS registry, 171 (13.7%) were aged below 70 years at enrollment (mainly men 164 [95.9%]). The study found that patients enrolled before 70 years of age tended to be more functionally impaired (17.1% with a Karnofsky score ≤70) and with more severe cardiac insufficiency (23.3% with a NYHA functional class III or IV).

### Patient characteristics

Clinically, the group of patients with an early diagnosis (ATTRwt-Yy group) differed substantially from those diagnosed after 65 years of age. Patients in the ATTRwt-Yy group at diagnosis had severe cardiopathy (based on the echocardiography parameters), as did patients in the ATTRwt-Oy and ATTRwt-Oo groups but presented with more tenosynovial and osteoarticular signs and symptoms. The increased incidence of tenosynovial and osteoarticular signs and symptoms in these patients suggests a specific phenotype or a diagnostic bias associated with a suspicion of ATTRwt-CM based predominantly on extracardiac signs and symptoms. Forty percent of the patients in the ATTRwt-Yy group had a CTS as first sign and symptom of ATTRwt-CM. Indeed, CTS is known to be associated with ATTRwt-CM, with CTS present 5 to 9 years before ATTRwt-CM diagnosis.^15^ The reported prevalence of CTS in patients with ATTRwt-CM ranges from 15% to 60%.^12^^,^^16^, ^17^, ^18^, ^19^, ^20^

### Prognosis

Our study demonstrates that ATTRwt-Yy patients exhibit better survival compared to older patient groups from the time of diagnosis. Nevertheless, despite their youthful age and relative to the general population and considering the natural history of ATTR-CM, the life expectancy of these patients may still be reduced. Thus, these patients may benefit from an early and intensified therapeutic strategy (including possible cardiac transplants) and associated with a reinforced follow-up. We have previously reported that cardiac transplantation may be a viable option in selected patients with ATTR-CM.^21^ A retrospective study reported a median age of 62 years at cardiac transplantation in the 14 patients with ATTR-CM (11 with ATTRwt-CM) transplanted between 1990 and 2020.^22^ At 5 years after transplantation, 90% of these patients were alive. Thus, younger patients diagnosed with ATTRwt-CM may benefit from a different therapeutic strategy compared to those diagnosed after 65 years of age.

A recent retrospective study assessed survival in patients with ATTRwt-CM treated with tafamidis according to the age at diagnosis.^23^ Similar survival benefits (median survival from diagnosis) were observed with tafamidis in patients diagnosed before 80 years of age and those diagnosed after the age of 80 years.

### Patients with ATTRwt-CM diagnosed after the age of 65 years but with early signs/symptoms associated to cardiac amyloidosis

In our study, we also identified a group of 111 patients (7.8% of the study population) diagnosed after the age of 65 years but with early signs and symptoms associated to CA. It must be noted that these early signs and symptoms may not necessarily be due to CA but may be associated with other comorbidities. These patients seem to have a specific profile: with early cardiac manifestation more frequently associated with rhythm disturbances and conductive events, but less frequently with heart failure and cardiac hypertrophy. Their first extracardiac manifestations were similar to those of the other group of elderly patients. The diagnosis in these patients may be delayed because their initial signs and symptoms are not those usually characteristic of patients with ATTRwt-CM (particularly focused on left ventricular wall thickness)^5^ or are not sufficiently severe to impact their everyday functioning to suspect ATTRwt-CM. ATTRwt-CM may be more readily suspected in younger patients with functional decline. Also, exercise intolerance which exacerbates functional decline is one of the most common complaints in patient with ATTRwt-CM.^24^

In our study, almost all patients with early signs and symptoms but diagnosed after 65 years had rhythm disorders. Interestingly, a recent study found that 19% of patients older than 65 years with a pacemaker had ATTRwt-CM.^25^ An increased awareness of rhythm disorders as a preliminary “red flag” for ATTRwt-CM and the early implication in the diagnostic pathway of cardiologists, with expertise in rhythm disturbances, may lead to earlier diagnosis of ATTRwt-CM in these patients. Early diagnosis is known to be associated with extended survival.^26^

### Appropriateness of the after 65-year age limit established for suspecting ATTRwt-CM

The current ESC guidelines suggest that CA should be suspected in individuals with increased wall thickness with either heart failure, aortic stenosis, or red flag signs/symptoms, particularly if aged older than 65 years.^5^^,^^6^ Our results suggest that the age cutoff of 65 years may be inappropriate considering that 4.7% of patients with ATTRwt, in our study, were diagnosed before the age of 65 years, with a further 7.8% having signs and symptoms associated with ATTRwt-CM before the age of 65 years but with a delayed diagnosis. This latest group therefore suggests that a substantial proportion of patients were underdiagnosed before the age of 65 years and that the proportion of patients in the younger cohort may have been even higher.

We also recommended that the importance of rhythm disorders as an earlier indication of ATTR-CM be emphasized in younger patients. Increased awareness of this patient population may result in early diagnosis and treatment with possible survival benefits.

The recognition of the distinct subgroup of patients (in the ATTRwt-Yy group) with early diagnosis should incite further research to identify the underlying mechanisms and potential innate or environmental risk factors associated with this early development of amyloidosis and its progression in these relatively young patients.^27^^,^^28^ A treatment specifically tailored for these patients would be appropriate.

### Study limitations

HEAR is an observation registry with the inherent limitations of an observational study. Our estimation of the proportion of early diagnosis of ATTRwt-CM may be underestimated. Indeed, most of the centers are following the guidelines and screening in patients before 65 years may be low. Furthermore, the early sign or symptom evoking ATTRwt-CM in patients diagnosed with ATTRwt-CM after 65 years of age may not have been related to amyloidosis but to another cardiovascular condition. If this is the case, then this could explain the delayed diagnosis observed in patients in the ATTRwt-Oy group.

## Conclusions

In the Amylo-Young study, 4.7% of patients were diagnosed at aged 65 years or younger with ATTRwt-CM. Despite their young age, these patients showed a similar cardiac phenotype compared to older patients, but with more severe osteo-tendinous disease. These patients have demonstrated a better survival after diagnosis compared with older patients. Physicians need to be aware of this distinct group of ATTRwt-CM patients with distinct disease characteristics and survival outcomes and adapt their diagnosis and care strategies.

Perspectives **COMPETENCY IN MEDICAL KNOWLEDGE:** We have identified a group of patients with wild-type transthyretin cardiac myopathy diagnosed before the age of 65 years with distinct disease characteristics. A greater awareness, adapted follow-up and care management is needed for these relatively young patients.

**TRANSLATIONAL OUTLOOK:** Disease evolution appears to differ in patients diagnosed with wild-type transthyretin cardiomyopathy before the age of 65 years. Further research is required to understand pathophysiological mechanisms associated with the disease evolution in this population.

## Funding support and author disclosures

Dr Guijarro has received consultancy fees from 10.13039/100006400Alnylam and 10.13039/100004319Pfizer. Dr Piriou has received honoraria, consulting fees, and support for attending meeting and/or travel from 10.13039/100006400Alnylam and 10.13039/100004319Pfizer. Dr Sauer has received honoraria from Bristol Myers Squibb and 10.13039/100004319Pfizer. Dr Roubille has received grants/contracts from 10.13039/100000046Abbott and Air Liquide; has received consulting fees from 10.13039/100000046Abbott, Air Liquide, 10.13039/100004326Bayer, and 10.13039/100004319Pfizer; has received honoraria and/or support for attending meetings/travel from 10.13039/100000046Abbott, Air Liquide, 10.13039/100006400Alnylam, 10.13039/100004325AstraZeneca, 10.13039/100004326Bayer, Boehringer, Bristol Myers Squibb, GSK, Implicity, MSD, Newcard, 10.13039/100004336Novartis, Novo Nordisk, 10.13039/100004319Pfizer, QuidelOrtho, 10.13039/100004339Sanofi, Servier, Vifor, and Zoll; has participated in a board for Carmat; has a leadership or fiduciary role in a board/committee/society for 10.13039/100001003Boehringer Ingelheim, 10.13039/100004336Novartis, and Vifor; and has received support for medical writing from 10.13039/100004319Pfizer. Dr Réant has received honoraria, consulting fees, and support for attending meetings/travel from 10.13039/100006400Alnylam and 10.13039/100004319Pfizer. Dr Donal has received grants from 10.13039/100006400Alnylam and consulting fees from 10.13039/100006400Alnylam, 10.13039/100004325AstraZeneca, and 10.13039/100004319Pfizer. Dr Lairez has received speaking fees from 10.13039/100006400Alnylam Pharmaceuticals, 10.13039/100015362Amicus Therapeutics, 10.13039/100004325AstraZeneca, BMS, Bridgebio, 10.13039/100004319Pfizer, Sanofi-Genzyme, and Siemens Healthiners; consulting fees from 10.13039/100006400Alnylam Pharmaceuticals, 10.13039/100015362Amicus Therapeutics, 10.13039/100004325AstraZeneca, and 10.13039/100004319Pfizer; and educational support to his institution from 10.13039/100004319Pfizer. Dr Courand has received consulting fees and honoraria (as an author) from 10.13039/100006483Abbvie, 10.13039/100004325AstraZeneca, 10.13039/100002429Amgen, Astellas, 10.13039/100001003Boehringer Ingelheim/10.13039/100004312Lilly, Bristol Myers Squibb, 10.13039/100004374Medtronic, 10.13039/100014369Organon, and 10.13039/100004319Pfizer. Dr Dagrenat has received grants, consulting fees, honoraria and/or support for attending meetings/travel from 10.13039/100006400Alnylam, 10.13039/100004325AstraZeneca, 10.13039/100001003Boehringer Ingelheim, Bristol Myers Squibb, and 10.13039/100004319Pfizer. Dr Gueffet has received honoraria and/or support for attending meetings or for travel from 10.13039/100000046Abbott, 10.13039/100004325AstraZeneca, 10.13039/100004326Bayer, 10.13039/100001003Boehringer Ingelheim, CMD Health, GSK, Janssen Cilag, 10.13039/100004374Medtronic, MSD, 10.13039/100004336Novartis, 10.13039/100004319Pfizer, 10.13039/100004339Sanofi, and Viatris. Dr Jeanneteau has received honoraria from 10.13039/100004325AstraZeneca, Bristil Myers Squibb, 10.13039/100004336Novartis, Novo Nordisk, and 10.13039/100004319Pfizer. Dr Oghina has received consulting fees and honoraria from 10.13039/100006400Alnylam, 10.13039/100004325AstraZeneca, 10.13039/100004326Bayer, and 10.13039/100004319Pfizer. Dr Damy has received research grant or consultancy fees from 10.13039/100006400Alnylam, 10.13039/100006396Alexion, 10.13039/100004325AstraZeneca, 10.13039/100004326Bayer, Bridge-Bio, 10.13039/100004319Pfizer, and Neurimmune. Dr Costa has received honoraria, consulting fees from Alnylam, Pfizer, Boehringer, Novartis, Servier, Amgen and Bristol Myers Squibb and support for attending meetings and/or travel from Novartis, Pfizer, Servier, Bristol Myers Squibb, AstraZeneca and Alnylam, has participated on a Data Safety Monitoring Board or Advisory Board for AstraZeneca, Pfizer, Novartis, Novonordisk and Sanofi Genzyme. All other authors have reported that they have no relationships relevant to the contents of this paper to disclose. HEAR is promoted by the HEART foundation.
